# Case report of ureteric transection of lower moiety in high-speed road traffic collision

**DOI:** 10.1093/jscr/rjab417

**Published:** 2021-09-30

**Authors:** Carol-Ann McCorgray, Senthil Ragupathy, Alexander Beaumont, Ismail El Mokadem, Sinan Khadhouri, Grigorios Athanasiadis

**Affiliations:** Department of Urology, Aberdeen Royal Infirmary, Aberdeen, Scotland, UK; Department of Radiology, Aberdeen Royal Infirmary, Aberdeen, Scotland, UK; Department of Radiology, Aberdeen Royal Infirmary, Aberdeen, Scotland, UK; Department of Urology, Aberdeen Royal Infirmary, Aberdeen, Scotland, UK; Department of Urology, Aberdeen Royal Infirmary, Aberdeen, Scotland, UK; Health Services Research Unit, University of Aberdeen, Aberdeen, Scotland, UK; Department of Urology, Aberdeen Royal Infirmary, Aberdeen, Scotland, UK

## Abstract

Ureteric injuries from blunt trauma are rare in adults requiring prompt diagnosis and management. To our knowledge this is the second case report of a complete transection of the ureter due to blunt injury at the pelvi-ureteric junction in an adult. Following a high-speed road traffic collision, a 26-year-old female with bilateral duplex kidneys was admitted with complete transection of the lower moiety of her right collecting system confirmed on computed tomography Urogram. This was repaired successfully with a minimally invasive laparoscopic technique in keeping with European Association of Urology guidelines. A 3-month follow-up MAG 3 renogram indicated adequate drainage from the right kidney with no evidence of obstruction. This successful outcome demonstrates a laparoscopic repair is achievable and favourable, improving post-operative recovery and reduction of inpatient length of stay. We recommend that a laparoscopic approach should be attempted unless laparotomy is indicated for other injuries.

## INTRODUCTION

Ureteric injuries from external trauma are rare in adults and usually involve penetrating injuries. Ureteric injuries from blunt trauma are equally rare. In children they usually occur in rapid deceleration and are associated with polytrauma and multiple organ injuries [1, 2]. The diagnosis and prompt management of these injuries is paramount to reduce long-term complications and morbidity. To our knowledge there has only been one case report of a complete transection of the ureter at the pelvi-ureteric junction in an adult (41-year-old man) following blunt trauma in a road traffic collision, which was successfully repaired via laparotomy [3].

## CASE REPORT

A 26-year-old lady was involved in a high-speed road traffic collision whilst driving during the evening. The vehicle, carrying four children and one other adult, hit an object on the road and veered off road into an embankment. The adult passenger was found dead at the scene.

She was taken to a local hospital and had a trauma computed tomography (CT) series showing fractures to the anterior right hemi-sacrum, posterior margin of right iliac bone extending to right anterior acetabular column, right inferior pubic ramus and right L2 transverse process. These were associated with pelvic bleeding and haematoma. She had abdominal right sided perinephric fluid and a ureteric injury could not be excluded.

She was resuscitated and stabilized with a pelvic binder and transferred to Aberdeen Royal infirmary for further management.

**Figure 1:**
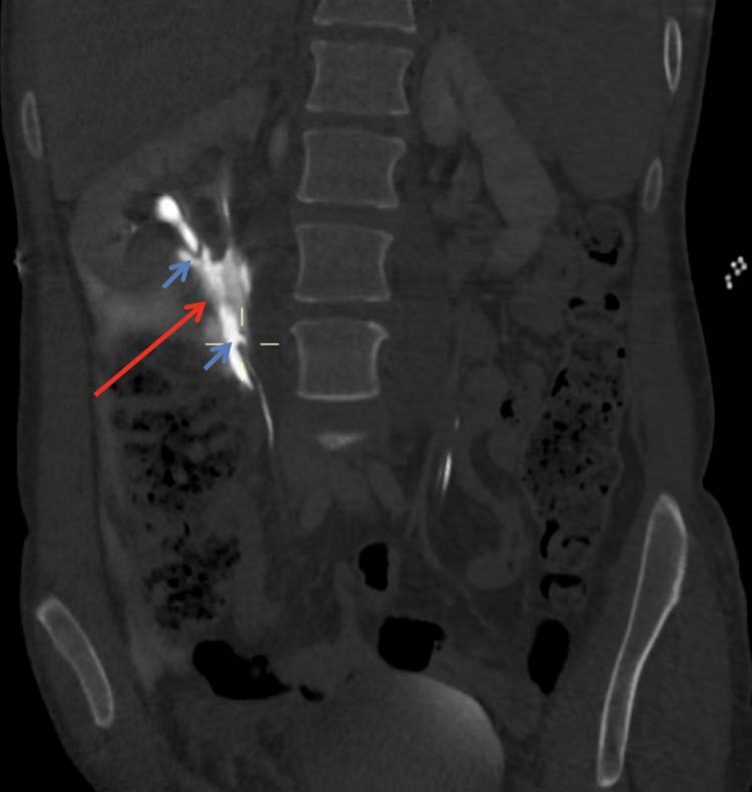
CT Urogram demonstrating extravasation of contrast. The blue arrows show the severed ends of the right inferior collecting system. The red arrow shows the contrast extravasation.

**Figure 2:**
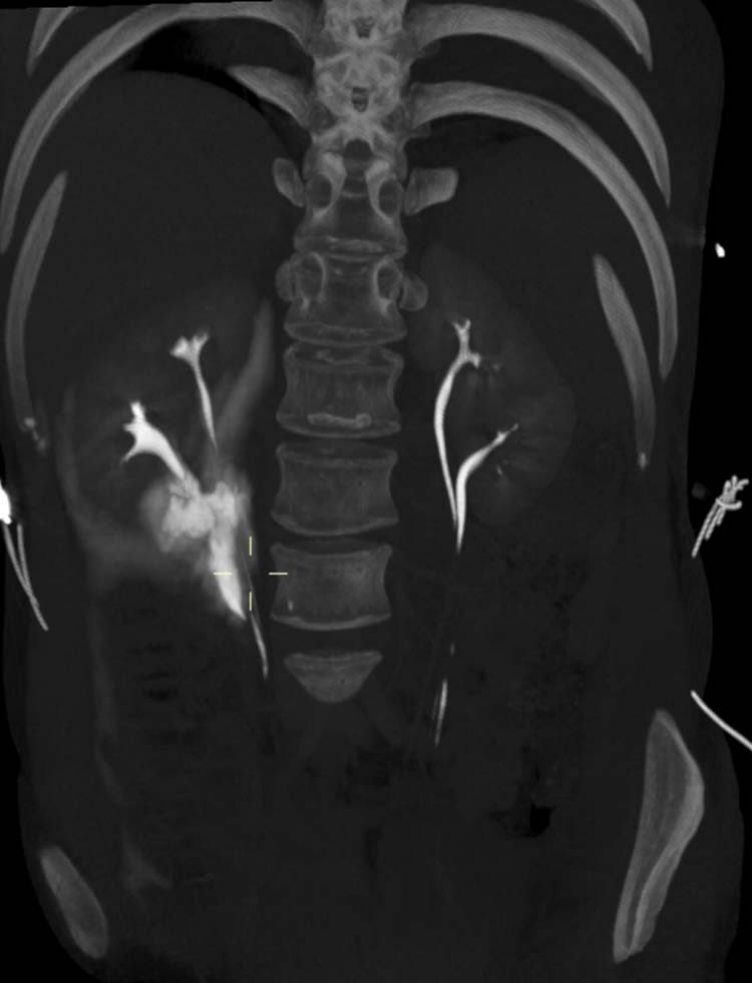
3D maximum intensity projection image of bilateral duplex kidneys and the contrast extravasation from the inferior moiety.

On arrival at 02:00, her temperature was 40.3°C with stable vital signs. After assessment by a multidisciplinary trauma team (orthopaedics, general surgery and urology), she had a CT Urogram that showed duplex kidneys and extravasation of contrast within the right peritoneum in keeping with urine leak (Figs 1 and 2). The lower moiety ureter was discontinuous with a mildly thickened and hyper-enhancing wall. The area of discontinuity was related to the highest density fluid on urographic phase, in keeping with this being the source of urine leak. The right upper moiety ureter appeared to be intact. There was no concomitant parenchymal injury.

The patient had a urinary catheter inserted and booked onto the emergency theatre list for cystoscopy +/− retrograde +/− ureteric stent insertion (right) +/− laparotomy +/− proceed. She was taken to theatre at 14:30 and had prophylactic intravenous Gentamicin 120 mg and Co-amoxiclav 1.2 g. Retrograde ureteropyelogram revealed a partially duplex system with an intact right upper moiety and extravasation of contrast from the right proximal lower moiety.

**Figure 3:**
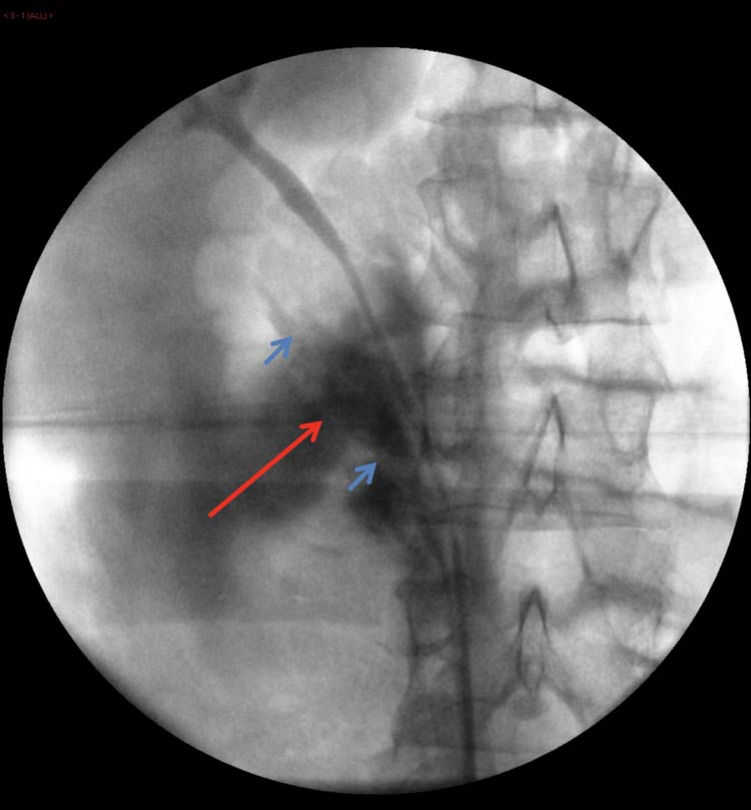
Retrograde ureteropyelography of right collecting system in theatre. The blue arrows show severed ends of the right inferior collecting system. The red arrow shows the contrast extravasation.

Following discussion with multiple consultant urologists, and in an attempt to avoid a potential laparotomy, a retrograde semi-rigid ureteroscopy was performed to try and stent the transected lower moiety ureter. This showed an intact lumen for ~10-cm proximal to the bifurcation up to an abrupt end in keeping with a transected ureter (Fig. 3). Beyond this, only fat was visualized and the proximal transected end was not seen. Endoscopic retrograde stenting was abandoned and the decision to convert to laparoscopic exploration was made. A 6Ch ureteric catheter was inserted to facilitate identification of the ureter before converting. 3 × 12-mm balloon ports were inserted (open/Hasson technique and direct visualization). A significant retroperitoneal collection was visualized, consistent with urinoma/irrigation fluid. The ascending colon and duodenum were mobilized and the inferior vena cava and gonadal vein were identified. The intact upper moiety ureter was identified. The distal end of the lower moiety was mobilized from the bifurcation and followed proximally to the level of injury where a complete transection was noted. Further dissection towards the kidney revealed the proximal end of the lower moiety. The two ends were spatulated and approximated. The lower moiety ureter was reconstructed using interrupted 4-0 vicryl and a 6Fr/26 cm JJ stent inserted over Sensor® guidewire. The retroperitoneum was washed out, the anastomosis covered with omentum, a 20Fr Robinson’s drain was inserted and port sites were closed.

**Figure 4:**
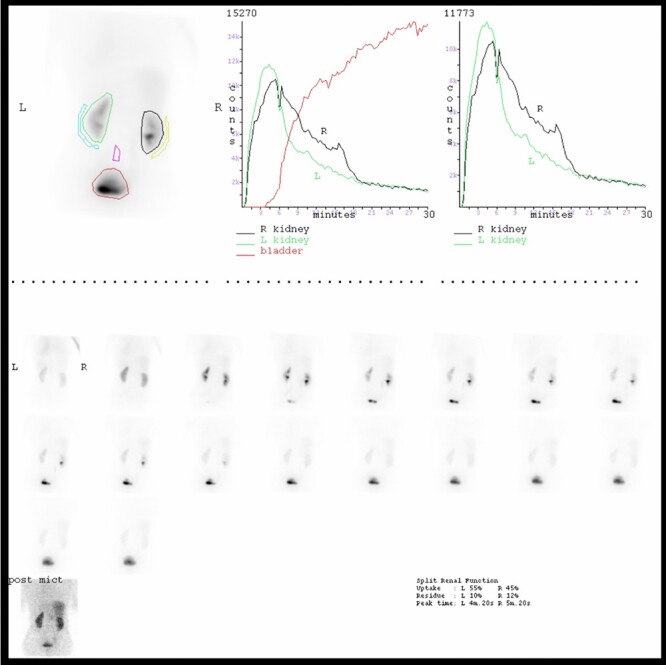
MAG 3 Renogram indicating slightly asymmetrical renal function (left better than right) and no evidence of true outflow obstruction but a mildly hypotonic right renal collecting system.

She was extubated at 21:30 and following recovery was transferred to high-dependency unit at 23:30. Her vital signs were stable with good urine output. She was reviewed the following morning and was noted to have 145 ml of haemoserous fluid from her drain post operatively. She was taken to theatre by orthopaedics on Day 2 post admission for internal fixation of her pelvic fractures with no complications. Her pelvic drain was removed on Day 3.

She continued to improve and was discharged from hospital on Day 11 with physiotherapy and rehabilitation. A flexible cystoscopy and removal of ureteric stent was carried out 4 weeks later. This was tolerated well although the distal coil of the stent was encrusted.

She was followed up with a Renogram 3 months later. This showed the right kidney had adequate tracer uptake and demonstrated slightly sluggish drainage, which responded to diuretic stimulus indicating no evidence of true outflow obstruction (Fig. 4). Relative renal function (right–left) was 45%:55%.

She has remained well and has had no further right flank pain or complications.

## DISCUSSION

To our knowledge, this is the second case report of a ureteric transection secondary to blunt trauma in an adult. Our case differs in complexity due to bilateral duplex ureters and by our successful minimally invasive laparoscopic ureteric repair.

We hypothesise the fracture of the right L2 transverse process may indicate the area of maximum impact. This could be due to the rapid deceleration from the lap seatbelt acting as a fulcrum, which led to the ureteric injury [3]. This was classified as a Grade 4 ureteric injury as per the American Association for the Surgery of Trauma [1].

The prompt diagnosis and management of the ureteric injury in our case was in line with the European Association of Urology (EAU) guidelines and led to a successful outcome with good drainage evident on the follow-up renogram. The repair was in keeping with all six principles of repair set out by EAU [1].

Furthermore, we have demonstrated that a successful repair can be achieved with minimally invasive surgery via laparoscopic approach, which improved post-operative recovery and reduced inpatient length of stay. We would therefore recommend a laparoscopic approach should be attempted initially for repair of blunt ureteric injuries unless a laparotomy is indicated for other intra-abdominal injuries.
